# Transcriptomic Analysis Reveals the Mechanism of Color Formation in the Peel of an Evergreen Pomegranate Cultivar ‘Danruo No.1’ During Fruit Development

**DOI:** 10.3390/plants13202903

**Published:** 2024-10-17

**Authors:** Xiaowen Wang, Chengkun Yang, Wencan Zhu, Zhongrui Weng, Feili Li, Yuanwen Teng, Kaibing Zhou, Minjie Qian, Qin Deng

**Affiliations:** 1 School of Breeding and Multiplication (Sanya Institute of Breeding and Multiplication), Hainan University, Sanya 572025, China; xiaowenwang@hainanu.edu.cn (X.W.); hndxyck@hainanu.edu.cn (C.Y.); wencanzhu@hainanu.edu.cn (W.Z.); zhongrui.weng@hainanu.edu.cn (Z.W.); feili.li@hainanu.edu.cn (F.L.); zkb@hainanu.edu.cn (K.Z.); 2 Key Laboratory of Quality Regulation of Tropical Horticultural Crop in Hainan Province, School of Tropical Agriculture and Forestry, Hainan University, Haikou 570228, China; 3 Hainan Institute of Zhejiang University, Sanya 572000, China; ywteng@zju.edu.cn

**Keywords:** *Punica granatum*, pigment, anthocyanin, carotenoid, chlorophyll, RNA-Seq

## Abstract

Pomegranate (*Punica granatum* L.) is an ancient fruit crop that has been cultivated worldwide and is known for its attractive appearance and functional metabolites. Fruit color is an important index of fruit quality, but the color formation pattern in the peel of evergreen pomegranate and the relevant molecular mechanism is still unknown. In this study, the contents of pigments including anthocyanins, carotenoids, and chlorophyll in the peel of ‘Danruo No. 1’ pomegranate fruit during three developmental stages were measured, and RNA-seq was conducted to screen key genes regulating fruit color formation. The results show that pomegranate fruit turned from green to red during development, with a dramatic increase in a* value, indicating redness and anthocyanins concentration, and a decrease of chlorophyll content. Moreover, carotenoids exhibited a decrease–increase accumulation pattern. Through RNA-seq, totals of 30, 18, and 17 structural genes related to anthocyanin biosynthesis, carotenoid biosynthesis and chlorophyll metabolism were identified from differentially expressed genes (DEGs), respectively. Transcription factors (TFs) such as MYB, bHLH, WRKY and AP2/ERF were identified as key candidates regulating pigment metabolism by K-means analysis and weighted gene co-expression network analysis (WGCNA). The results provide an insight into the theory of peel color formation in evergreen pomegranate fruit.

## 1. Introduction

Pomegranate (*Punica granatum* L.) is an ancient deciduous fruit crop that originated from the area of Iran to the Himalayan Mountains in northern India, and is cultivated in many subtropical and temperate regions around the world [1]. It is a kind of excellent fruit tree that combines ecological and ornamental values with health care functions [2]. There are 30 evergreen pomegranate cultivars in Punjab India, and ‘Vietnamese’ is an evergreen cultivar from Vietnam [3]. ‘Danruo No. 1’ is an evergreen pomegranate cultivar bred in 2019, and is the only commercial cultivar cultivated in the tropical region of China. Fruit color is a crucial index of fruit appearance quality, which can serve as an indicator for assessing food quality and grade [4], thereby influencing consumers’ preferences and the commercial value of the products. For example, consumers exhibit a pronounced preference for apples [5], peaches [6], and Chinese cherries [7] with uniform coloration and a predilection for red hues. The pigmentation of fruit primarily derives from the concentration and proportional distribution of three key pigments: anthocyanins, carotenoids, and chlorophyll [8].

Anthocyanins are water-soluble pigments belonging to flavonoids class, generating red, blue and purple colors in the flowers and fruits [9]. Anthocyanins are synthesized through the phenylpropanoid and flavonoid pathways, and the involved enzymes include phenylalanine ammonia-lyase (PAL), cinnamate 4-hydroxylase (C4H), 4-coumarate-CoA ligase (4CL), chalcone synthase (CHS), chalcone isomerase (CHI), flavanone 3-hydroxylase (F3H), flavonoid 3′-hydroxylase (F3′H), flavonoid 3′,5′-hydroxylase (F3′5′H), dihydroflavonol 4-reductase (DFR), anthocyanidin synthase (ANS), and UDP-glucose: flavonoid 3-O-glucosyltransferase (UFGT) [9]. Genes encoding these proteins are called structural genes. Anthocyanins are then transferred to vacuoles for storage, and the enzyme involved in this process is probably glutathione *S*-transferase (GST) [10]. The expression of structural genes is regulated by transcription factors (TFs), and MYB, bHLH and WD40 are the predominant TFs that can form a complex and participate in the regulation of anthocyanins biosynthesis [9].

Carotenoids constitute a significant class of natural pigments, and plant tissues or organs accumulating carotenoids exhibit orange, yellow or red colors. The carotenoids biosynthesis pathway has been clarified in diverse plant species [11]. Carotenoids are predominantly synthesized via the plastid-localized methylerythritol 4-phosphate (MEP) pathway in plants, which generates the precursor of carotenoids, i.e., geranylgeranyl diphosphate (GGPP) [11]. GGPP is then converted to the first colorless carotenoid 15-cis-phytoene catalyzed by phytoene synthase (PSY). PSY serves as a crucial rate-limiting enzyme of carotenoid biosynthesis [12,13]. Phytoene is subsequently desaturated and isomerized to produce red-colored lycopene catalyzed by phytoene desaturase (PDS), ζ-carotene isomerase (ZISO), ζ-carotene desaturase (ZDS), and carotenoid isomerase (CRTISO). Lycopene is cyclized by lycopene ε-cyclase (LCYE) and/or lycopene β-cyclase (LCYB) to produce orange-colored α-carotene and β-carotene, respectively. The hydroxylation of α- and β-carotene is catalyzed by two cytochrome P450-type hydroxylases (CYP97A and CYP97C) and two non-heme β-ring hydroxylases (BCH1 and BCH2) to form yellow-colored lutein and zeaxanthin, respectively. Zeaxanthin is catalyzed by zeaxanthin epoxidase (ZEP) to produce violaxanthin and reversed back by violaxanthin de-epoxidase (VDE), which is the called xanthophyll cycle. Violaxanthin is transformed into neoxanthin by neoxanthin synthase (NSY). Violaxanthin and neoxanthin are catalyzed by 9-cis-epoxycarotenoid dioxygenase (NCED) to yield abscisic acid (ABA).

The chlorophyll molecule mainly consists of a porphyrin ring containing a magnesium atom and a long carbon side chain. The chlorophyll metabolic pathway can be classified into three phases [14,15]. The first phase involves the synthesis of chlorophyll a from glutamate, and enzymes participating in this process include glutamyl-tRNA synthetase (GluRS), glutamyl-tRNA reductase (HEMA), glutamate-1-semialdehyde 2,1-aminotransferase (GSA), 5-aminolevulinate dehydrogenase (HEMB), porphobilinogen deaminase (HEMC), uroporphyrinogen III synthase (HEMD), uroporphyrinogen III decarboxylase (HEME), coproporphyrinogen III oxidase (HEMF), protoporphyrinogen oxidase (HEMG), Mg-chelatase (CHLH/D/I), Mg-protoporphyrin IX methyltransferase (CHLM), Mg-protoporphyrin IX monomethylester cyclase (CRD), protochlorophyllide oxidoreductase (POR), divinyl chlorophyllide a 8-vinyl-reductase (DVR), and chlorophyll synthase (CHLG/P). The second phase encompasses the interconversion of chlorophyll a and chlorophyll b, named the chlorophyll cycle, and geranylgeranyl-diphosphate reductase (CAO), chlorophyllide a oxygenase (NYC1/NOL), and chlorophyll b reductase (HCAR) are the key enzymes involved in this process. The third phase includes the degradation of chlorophyll a, which includes hydroxymethyl chlorophyll a reductase (CLH), magnesium dechelatase (MCS), pheophytin phephorbide hydrolase (PPH), pheophorbide a oxygenase (PaO), and red chlorophyll catabolite reductase (RCCR).

Fruit color is largely associated with fruit development. In pear, chlorophyll content is decreased during fruit development in both occidental and oriental pears, while anthocyanins content shows a decrease pattern in occidental pears and an increase–decrease pattern in oriental pears [16]. In pomegranate, based on RNA-seq, the expression patterns of anthocyanin biosynthetic genes in aril and fruit peels at different developmental stages have been analyzed in the deciduous cultivars ‘Taishanhong’ and ‘Dabenzi’ [17,18]. During fruit ripening, total chlorophylls and carotenoids contents in ‘Taishanhong’ pomegranate peel are decreased, while anthocyanin content shows a continuous increase pattern [19]. Anthocyanin content in the peel of ‘Moshiliu’ pomegranate shows two peaks during fruit development, which are detected at the early and late developmental stages, respectively, while ‘Hongbaoshi’ shows an additional peak at the middle of the developmental stage [20]. However, fruit coloration and the accumulation pattern of different pigments in the fruit peel of evergreen pomegranate cultivar during fruit development has not been assessed. According to previous studies, our hypothesis is that the chlorophyll content in evergreen pomegranate might also decrease during fruit development, while carotenoids and anthocyanin could show a unique pattern.

In this study, in order to comprehensively analyze the formation of fruit color in an evergreen pomegranate cultivar ‘Danruo No. 1’ and the relevant molecular mechanism, fruit peel at three developmental stages was sampled and the contents of anthocyanins, carotenoids, and chlorophyll were measured. RNA-seq was conducted to identify key structural genes and transcription factors related to color formation. This research will provide new insights into the peel color formation mechanism in evergreen pomegranate.

## 2. Results

### 2.1. Coloration and Pigments Contents in ‘Danruo No.1’ Pomegranate Peel During Fruit Development

**Figure 1:**
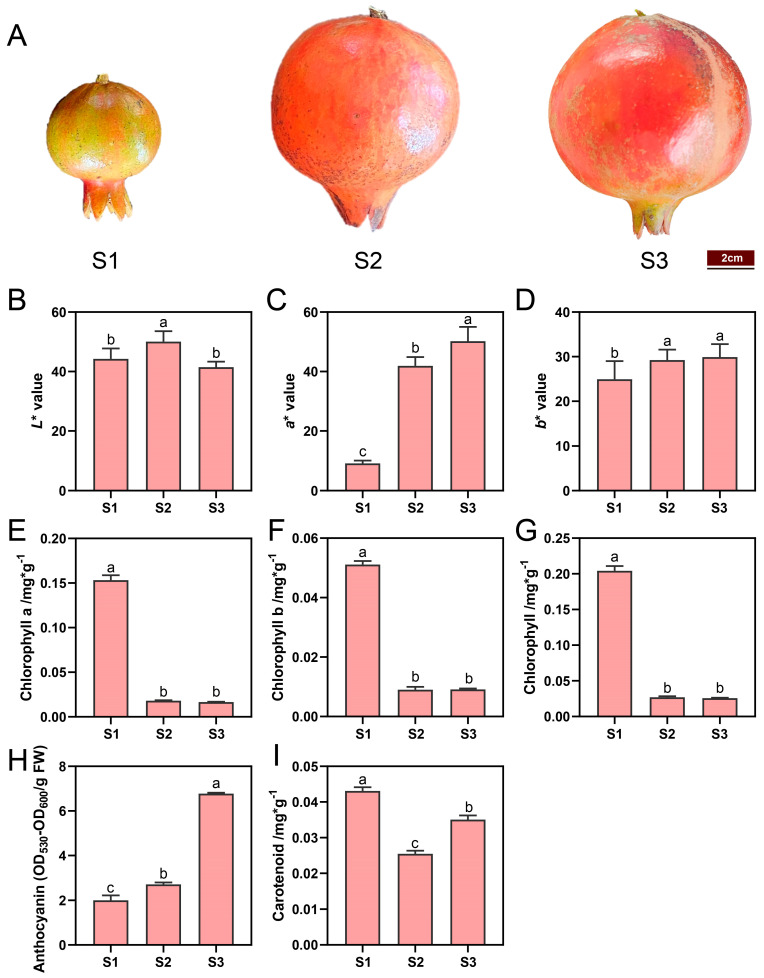
Coloration and pigment contents in ‘Danruo No.1’ pomegranate peel during fruit development. (**A**) Representative images of fruits at developmental stage 1 (S1), stage 2 (S2), and stage 3 (S3). (**B**) Fruit peel lightness (L* value). (**C**) Fruit peel a* value (higher value means redness and lower value means greenness). (**D**) Fruit peel b* value (higher value means yellowness and lower value means blueness). (**E**) Chlorophyll a content. (**F**) Chlorophyll b content. (**G**) Total chlorophyll content. (**H**) Anthocyanin content. (**I**) Carotenoid content. Each value represents the mean ± standard deviation of three biological replicates. Different letters above the bars indicate significant differences (*p* < 0.05) according to one-way analysis of variance (ANOVA) followed by Tukey test.

The peel coloration of ‘Danruo No.1’ pomegranate during fruit development is shown in Figure 1A. The fruit peel was green at stage 1 (S1), but turned to red at stage 2 (S2) and stage 3 (S3). The L* value representing lightness in peel showed an increase–decrease patter, with the peak detected at S2, reaching the value of 50.06 (Figure 1B). The a* value in peel increased constantly during fruit development, reaching the highest value of 50.23 at S3 (Figure 1C). Peel b* values at S2 and S3 were significantly higher than those at S1, but there was no significant difference between S2 and S3 (Figure 1D).

The contents of chlorophyll a, chlorophyll b and total chlorophyll in pomegranate peel peaked at S1, but showed very low levels at S2 and S3, and there was no significant difference between these two stages (Figure 1E–G, Table S1). The total anthocyanin content in pomegranate peel increased significantly with fruit development (Table S1). The total anthocyanin content of S3 was 3.39 times and 1.36 times higher than that of S1 and S2, respectively (Figure 1H). Carotenoid content decreased first and then increased, and the content at S3 was still significantly lower than at S1 (Figure 1I, Table S1).

### 2.2. RNA-seq Data Overview

A total of 42.34–57.38 million raw reads were obtained by the double-end sequencing of nine samples, resulting in a total of 41.70–55.07 million clean reads and a 6.25–8.26 G clean base (Table 1). The overall error rates were not more than 0.03%, the quality scores of 20 (Q20) values were around 98%, and the Q30 values were higher than 93.8% (Table 1). The GC contents ranged from 49.81% to 50.94% (Table 1). Principal component analysis (PCA) showed that PC1 and PC2 accounted for 49.82% and 23.52% of the total variation, respectively (Figure S1). Samples from three biological replicates of the same developmental stage clustered together, while samples from different developmental stages showed clear separation (Figure S1).

### 2.3. Differentially Expressed Genes (DEGs) Identification, and Gene Ontology (GO) and Kyoto Encyclopedia of Genes and Genomes (KEGG) Analyses

**Figure 2:**
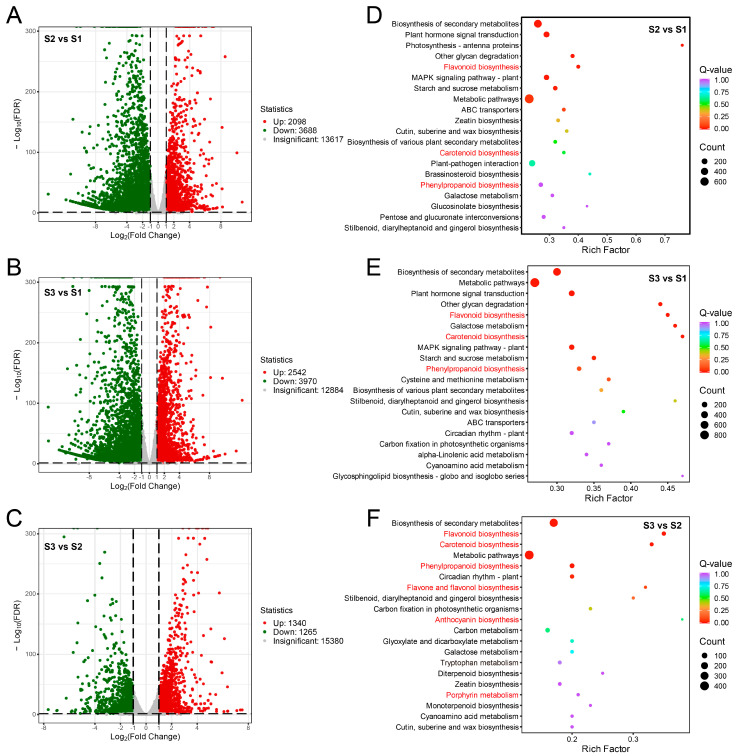
Differentially expressed genes (DEGs) identification and KEGG analysis. Volcano plots of DEGs from S2 vs. S1 (**A**), S3 vs. S1 (**B**), and S3 vs. S2 (**C**). Horizontal coordinates indicate the fold change of gene expression between different groups, and vertical coordinates indicate the significance level of gene expression difference in the two groups. Red dots indicate upregulated genes, green dots indicate downregulated genes, and grey dots indicate insignificant genes. Top 20 metabolic pathways analyzed by KEGG enrichment for DEGs from S2 vs. S1 (**D**), S3 vs. S1 (**E**), and S3 vs. S2 (**F**). The pathways associated with pigments metabolism are highlighted in red color.

According to the DEGs multiple screening (log2|FoldChange| > 1) and significant false discovery rate (FDR) < 0.05, totals of 2098, 2542, and 1340 up-regulated DEGs were detected in the comparison groups of S2 vs. S1, S3 vs. S1, and S3 vs. S2, respectively, and the corresponding numbers of downregulated DEGs were 3688, 3970, and 1265, respectively (Figure 2A–C).

The results of GO analyses of DEGs are shown in Figure S2. The DEGs were mainly annotated into cellular component, molecular function and biological process categories, which can be further divided into 40 subgroups. For DEGs from S2 vs. S1, the highest numbers of genes were annotated to secondary metabolism (159) in biological process, cytoskeleton (145) in cellular component, and tetrapyrrole binding (156) in molecular function, respectively. For S3 vs. S2, most DEGs were annotated to secondary metabolism (88) in biological process, plastid thylakoid (109) and chloroplast thylakoid (109) in cellular component, and oxidoreductase activity (95) and tetrapyrrole binding (95) in molecular function, respectively. For S3 vs. S1, most DEGs were annotated to secondary metabolism (180) in biological process, plastid thylakoid (186) and chloroplast thylakoid (186) in cellular component, and oxidoreductase activity (171) in molecular function, respectively.

KEGG analysis showed that most DEGs were annotated to metabolic pathway and the biosynthesis of secondary metabolites (Figure 2D–F). Regarding the pathways relevant to fruit coloration, flavonoid, carotenoid and phenylpropanoid biosynthesis pathways enriched DEGs from S2 vs. S1, S3 vs. S1, and S3 vs. S2 (Figure 2D–F). Besides this, DEGs from S3 vs. S2 were also enriched in flavone and flavonol biosynthesis, anthocyanin biosynthesis and porphyrin metabolism (Figure 2F).

### 2.4. DEGs Associated with Anthocyanin Biosynthesis in Pomegranate Peel During Fruit Development

**Figure 3:**
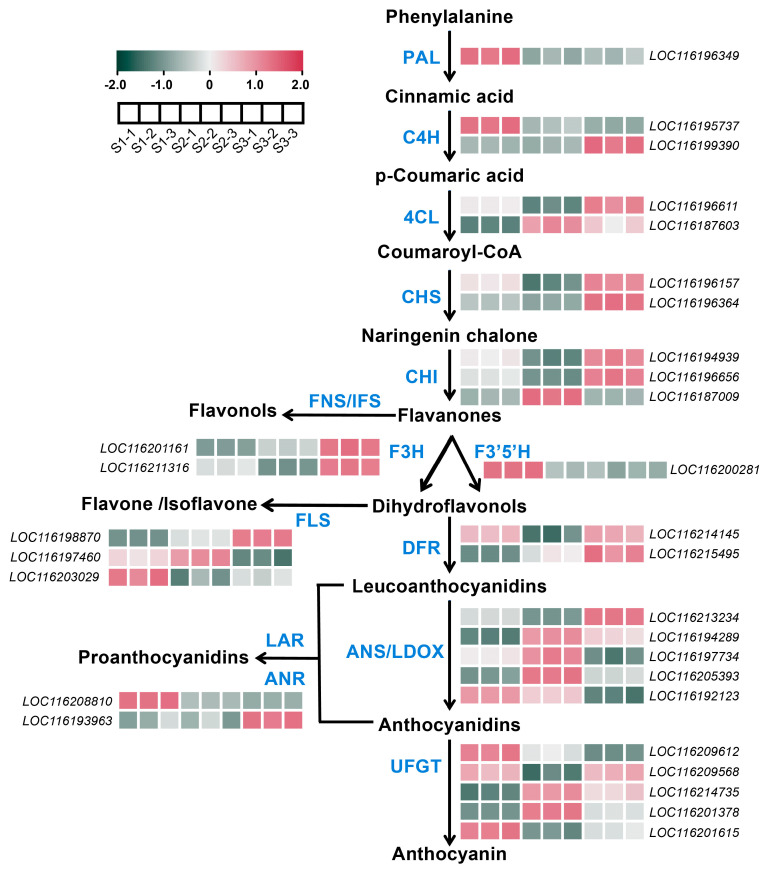
Expression patterns of the DEGs involved in anthocyanins synthesis in pomegranate peel at developmental stage 1 (S1), stage 2 (S2), and stage 3 (S3). The color scale from green to red represents the fragments per kilobase of transcript per million of fragments mapped (FPKM) values, from low to high.

A total of 30 DEGs involved in the anthocyanin biosynthetic pathway were identified (Table S2). Among them, genes including one *PAL*, one *C4H* (LOC116195737), one *F3′5′H*, one *ANS* (LOC116192123), one *FLS* (LOC116203029), one *ANR* (LOC116208810) and two *UFGTs* (LOC116209612 and LOC116201615) showed the highest expression levels at S1, with very low expression levels at S2 and S3 (Figure 3, Table S1). The expressions of one *DFR* (LOC116214145) and one *UFGT* (LOC116209568) showed a decrease–increase pattern, i.e., a high expression level was detected at S1 and S3 (Figure 3, Table S1). Most structural genes including one *C4H* (LOC116199390), two *4CLs*, two *CHSs*, two *CHIs* (LOC116196656 and LOC116187009), two *F3Hs* (LOC116201161 and LOC116211316), two *FLSs* (LOC116198870 and LOC116197460), one *DFR* (LOC116215495), four *ANSs* (LOC116213234, LOC116194289, LOC116197734, and LOC116205393), one *ANR* (LOC116193963), and two *UFGTs* (LOC116214735 and LOC116201378) showed a relatively higher expression level at S2 or S3, or both S2 and S3, when anthocyanin was highly accumulated (Figure 3, Table S1).

### 2.5. DEGs Associated with Carotenoids Biosynthesis in Pomegranate Peel During Fruit Development

**Figure 4:**
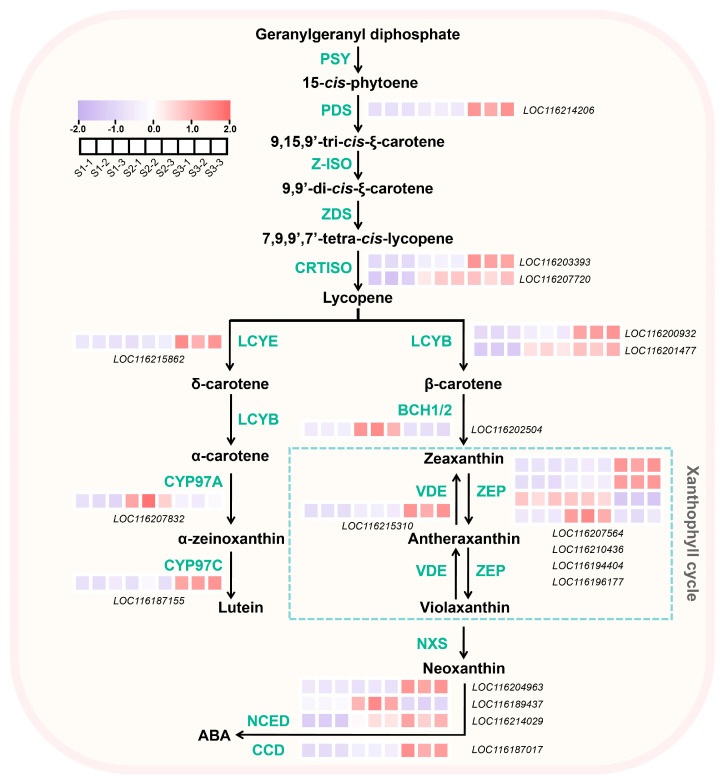
Expression pattern of the DEGs involved in carotenoids synthesis in pomegranate peel at developmental stage 1 (S1), stage 2 (S2), and stage 3 (S3). The color scale from blue to red represents the fragments per kilobase of transcript per million of fragments mapped (FPKM) values from low to high.

A total of 18 DEGs involved in the carotenoids biosynthetic pathway were identified (Table S2). One *ZEP* (LOC116194404) was highly expressed at S1 and S2 (Figure 4, Table S1). The expressions of one *PDS*, one *CRTISO* (LOC116203393), one *LCYB* (LOC116200932), one *VDE*, two *ZEPs* (LOC116207564 and LOC116210436), one *CCD* (LOC116187017) and one *NCED* (LOC116204963) showed the highest level at S3 (Figure 4, Table S1). For one *CYP97A*, one *CHYB*, one *ZEP* (LOC116196177), and one *NCED* (LOC116189437), relatively higher expression levels were detected only at S2 (Figure 4). In addition, the expressions of genes including one *CRTISO* (LOC116207720), one *LCYB* (LOC116201477), and one *NCED* (LOC116214029) were upregulated both at S2 and S3 (Figure 4, Table S1).

### 2.6. DEGs Associated with Chlorophyll Biosynthesis and Degradation in Pomegranate Peel During Fruit Development

**Figure 5:**
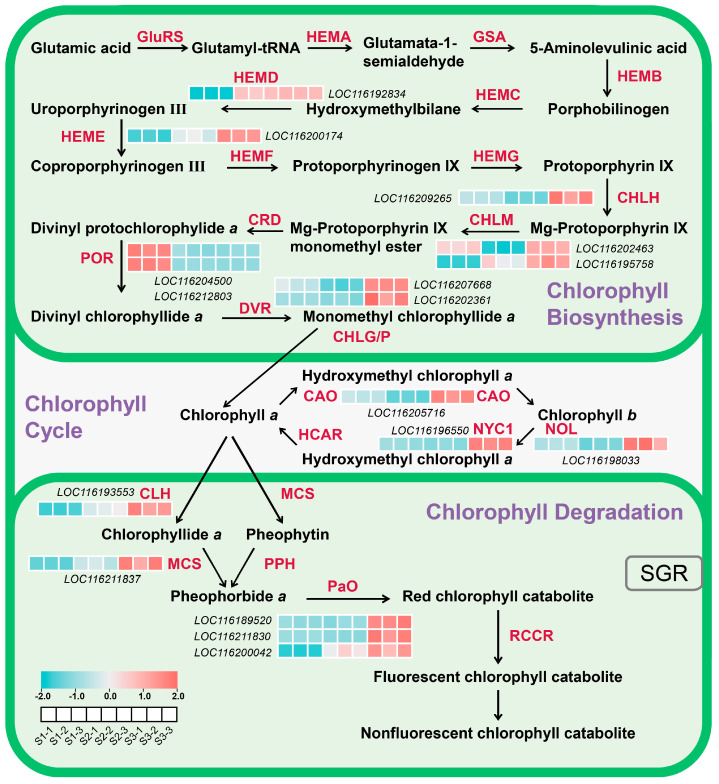
Expression pattern of the DEGs involved in chlorophyll biosynthesis and degradation in pomegranate peel at developmental stage 1 (S1), stage 2 (S2), and stage 3 (S3). The color scale from green to red represents the fragments per kilobase of transcript per million of fragments mapped (FPKM) values from low to high.

A total of 17 DEGs involved in chlorophyll biosynthesis and degradation were identified (Tables S1 and S2). Among them, *POR* was the only gene that was highly expressed at S1, and it decreased to a very low level at S2 and S3 (Figure 5, Table S1). Higher expression levels of one *CHLM* (LOC116202463) and one *HEMD* (LOC116192834) were detected at S1 and S3, and S2 and S3, respectively (Figure 5, Table S1). All the rest of the chlorophyll biosynthetic genes, including one *HEME*, one *CHLH*, one *CHLM* (LOC116195758), two *DVRs*, and one *CAO*, and degradative genes including one *CLH*, one *NYC1*, one *NOL*, one *MCS*, and three *PAOs*, were only highly expressed at S3 (Figure 5, Table S1).

### 2.7. Screening Pigments Metabolism-Related TFs Based on K-Means and Weighted Gene Co-Expression Network Analysis (WGCNA)

**Figure 6:**
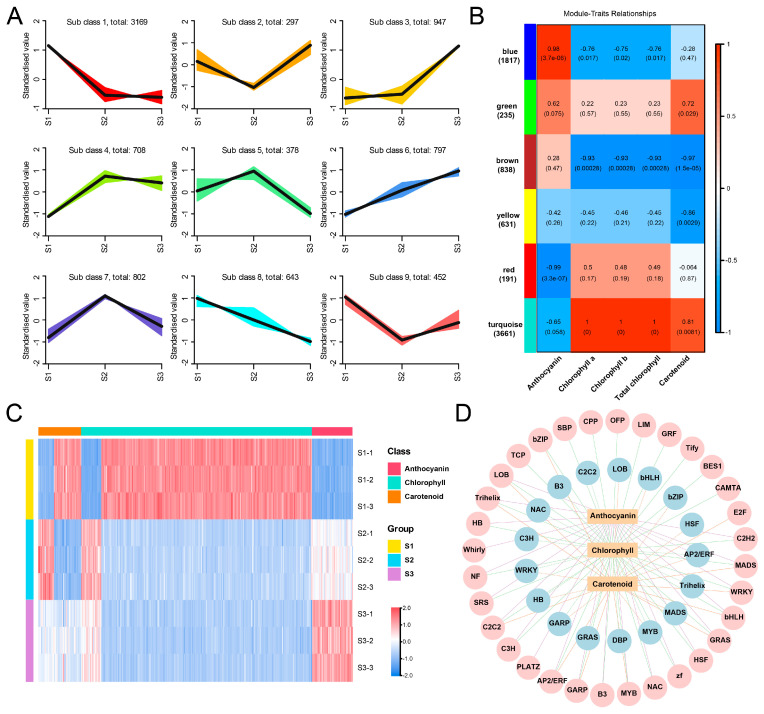
Identification of transcription factors (TFs) regulating pigments metabolism in pomegranate peel during fruit development. (**A**) K-means analysis of DEGs identified from transcriptome sequencing. The expression profiles of genes in each cluster are represented in different colors, and the average expression levels of all genes in developmental stage 1 (S1), S2, and S3 are represented in black. (**B**) Weighted gene co-expression network analysis (WGCNA) of DEGs identified from transcriptome sequencing. Module-trait correlations and corresponding *p*-values in parentheses. The left panel shows the six modules with gene numbers. The color scale on the right shows the module-trait correlations from −1 (blue) to 1 (red). ‘Anthocyanin’, ‘Chlorophyll a’, ‘Chlorophyll b’, ‘Total chlorophyll’ and ‘Carotenoid’ represent the changes in corresponding substances’ concentrations. (**C**) Heatmap presenting the expression patterns of regulatory genes regulating pomegranate peel pigments metabolism during fruit development. (**D**) Correlation network between TFs’ expression and pigments’ contents; pink and blue circles represent positive and negative correlations, respectively. Purple, orange, and green lines representing the relation between TFs and anthocyanin, carotenoid, and chlorophyll, respectively.

We followed a two-step approach to screening regulatory genes that potentially participate in pigment metabolism. Firstly, K-means clustering analysis was performed for all DEGs (Figure 6A). Based on K-means analysis, genes in clusters 6/8, clusters 1/4, and clusters 9/7 were regarded as candidate genes positively/negatively involved in the accumulation of anthocyanins, chlorophyll, and carotenoids, respectively (Figure 6A). Secondly, WGCNA was conducted to analyze the correlation between DEGs and pigments concentration. All the DEGs were clustered into six modules, and the blue and red modules exhibited strong positive and negative correlations with anthocyanins content, respectively (Figure 6B). Genes in the turquoise module showed an extremely positive correlation coefficient with chlorophyll content, and these were also positively correlated to carotenoids content, while genes in the brown module displayed a strong negative correlation with these two substances (Figure 6B). In addition, genes from the yellow module were also negatively correlated with carotenoids content (Figure 6B).

By integrating the results from the K-means and WGCNA analyses, 45/1, 30/16, and 231/22 regulatory genes (encoding TFs) were identified as key candidate genes that positively/negatively regulate the accumulation of anthocyanins, carotenoid, and chlorophyll, respectively (Table S3). The expressions of these genes are shown in Figure 6C. For anthocyanin, the positive regulators were from 21 TF families including MYB, WRKY, bHLH, GRAS, etc., and an AP2/ERF TF was the only identified negative regulator (Figure 6D). For carotenoid, the positive regulators were from 12 TF families mainly including MYB, C2C2, AP2/ERF, bZIP, and LOB, and the negative regulators were from 10 families, mainly including HB, MYB, GARP, and HSF (Figure 6D). Furthermore, 29 and 13 TF families were regarded as positive and negative regulators for chlorophyll accumulation, respectively, which both contain MYB, C2H2, AP2/ERF, LOB, TCP, and Trihelix (Figure 6D).

### 2.8. Validation of DEGs by Quantitative Polymerase Chain Reaction (q-PCR)

**Figure 7:**
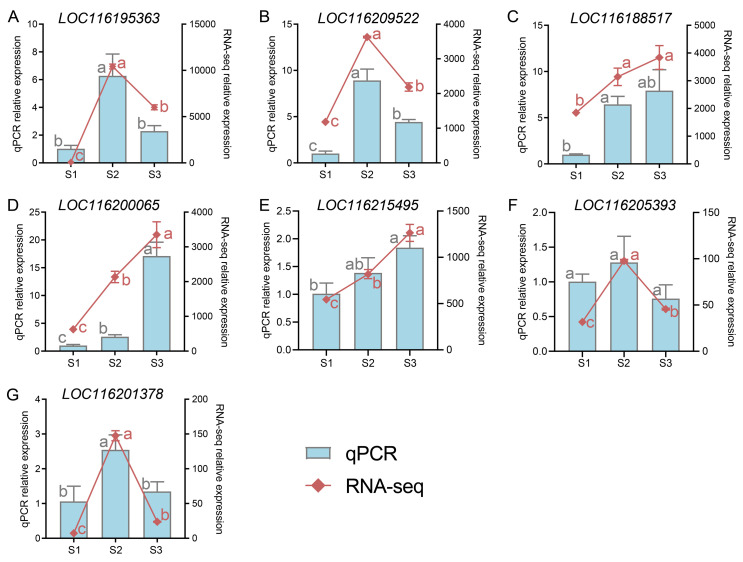
The expressions of seven genes in pomegranate peel at developmental stage 1 (S1), S2, and S3 from transcriptome data were examined by quantitative polymerase chain reaction (q-PCR). The expression levels obtained by RNA-seq and q-PCR are shown with a line chart and histogram, respectively. Data are presented as the mean ± standard deviation of three biological replicates. Different letters above the bars indicate significant differences (*p* < 0.05) according to one-way analysis of variance (ANOVA) followed by Tukey test. Data analyzed by qPCR (marked with gray letters) or RNA-seq (marked with red letters) were tested separately.

Seven DEGs were chosen and analyzed by q-PCR to confirm the accuracy of RNA-seq data. The results show that gene expression in pomegranate peel during fruit development by q-PCR was consistent with the RNA-seq results (Figure 7, Table S1).

## 3. Discussion

Fruit color is an important index for judging fruit quality and maturity, in which chlorophyll, carotenoid and anthocyanin determine the color of plant tissue. In this study, with the growth and development of the fruit, anthocyanins content was significantly upregulated, carotenoids content was decreased first and then increased slightly, and chlorophyll content continuously declined, leading to the fruit peel color consequently turning from green to red (Figure 1E–I, Table S1). The trend of anthocyanin content in ‘Danruo No. 1’ pomegranate peel is similar to that in ‘Taishanhong’ pomegranate, mulberry and mango [19,21,22], but different from that in ‘Moshiliu’ and ‘Hongbaoshi’ pomegranate, pear and grape, in which the peak of anthocyanin content is observed at the early or middle developmental stages [16,20,23]. The trend of carotenoid content in ‘Danruo No. 1’ pomegranate peel is different from that of ‘Taishanhong’ pomegranate, in which carotenoid content is continuously decreased during fruit development [19]. A decrease in chlorophyll content during fruit development was also seen in ‘Taishanhong’ pomegranate [19], pear [16], and kiwi fruit [24]. All these results indicate that chlorophyll degradation during fruit development is quite conserved in fruits, while the anthocyanin and carotenoid accumulation patterns are diverse among different plant species, even among different cultivars of the same species.

Anthocyanin biosynthetic genes including *CHS*, *CHI*, *F3H*, *DFR*, *ANS*, and *UFGT* showed a relatively higher expression level at late developmental stages (Figure 3), which is associated with the anthocyanin content (Figure 1H). *CHI* and *CHS* are considered two crucial enzyme genes in the flavonoid biosynthesis pathway, closely related to fruit coloration, and their expression patterns increase with fruit development [25]. In pansy, the expression of *CHS* is closely associated with anthocyanin production [26]. It has been reported that *PgCHS*, *PgF3H*, and *PgDFR* isolated from pomegranates have higher expression levels in red fruits, and their transcriptional levels increase synchronously with flavonoid accumulation [27]. LDOX and UFGT catalyze the final two steps in the anthocyanin synthesis pathway. In the study, both *LDOX* and *UFGT* exhibited high levels of expression during the fruit color-changing period (Figure 3), which was consistent with the corresponding expression patterns observed in apple and jujube fruit [28,29]. In Arabidopsis, the ANS-deficient mutants are sensitive to high light due to impaired anthocyanin biosynthesis [30]. In grapes, *UFGT* is found to be expressed only in the skins of colored grape varieties after the color-changing period, but not in white varieties or pulp, with upregulated expression during the color-changing period [31]. The expression level of *UFGT* in wild pomegranates is in concurrence with the accumulation of anthocyanins observed in the leaves, flowers, green, and red fruits of the pomegranate plant [32]. *AcUFGT3* serves as a crucial gene that oversees the biosynthesis and accumulation of anthocyanins in red meat kiwifruit [33]. All these results suggest that the upregulation of anthocyanin biosynthetic genes is necessary for anthocyanin accumulation and red coloration.

Carotenoids content showed a decrease–increase pattern (Figure 1I). Pomegranate fruits undergo cell division and expansion periods from S1 to S2, probably resulting in a dilution effect and a decreased trend of carotenoids content from S1 to S2. Carotenoids biosynthesis is usually associated with fruit ripening, causing an increased trend of carotenoids content from S2 to S3. Most carotenoid biosynthetic genes, as well as the degradation genes, showed the expression peak at S2 or S3 (Figure 4), indicating that the relatively lower degradation rate at S1 and higher biosynthesis rate at S3 might be responsible for the higher carotenoids contents at S1 and S3, respectively. In celery, the expression of *AgCCD4* is negatively correlated with carotenoid content [34]. *PpCCD4* shows different expression patterns in different varieties of peaches, and its expression level is significantly higher in white-flesh peaches than in yellow-flesh peaches [35]. In mango, the expression of carotenoid biosynthesis-related genes such as *PSY*, *ZDS*, *CHYB* and *ZEP* is positively correlated with total carotenoid content [36,37]. In addition, the overexpression of the MADS transcription factor CsMADS6 in citrus healing tissues induces the expression of *LCYb1*, *PSY*, *PDS*, and *CCD1*, which increases carotenoid contents [38]. In tobacco, silencing the *PDS* gene resulted in a decrease in total carotenoid content [39]. The overexpression of *IbLCYB2* and *IbCCD4* genes increases and decreases carotenoid content in sweet potato, respectively [40,41]. Studies have shown that the downregulation of the *ZEP* gene in potato not only increases the accumulation of zeaxanthin in tubers, but also increases the total carotenoid level by 5.7-fold compared to the control group [42].

Chlorophyll content showed a peak at S1, but very low levels at S2 and S3 (Figure 1E–G), and most genes involved in chlorophyll biosynthesis and degradation, as well as cycling, showed the highest expression at S3, with *POR* showing the highest expression level at S1 (Figure 5), indicating that a high expression level of *POR* and lowly expressed degradative genes might contribute to high chlorophyll content at S1, and highly expressed degradative genes are responsible for the low chlorophyll content at S3. In Arabidopsis, different *POR* members have divergent roles in regulating chlorophyll biosynthesis, with *PORB* and *PORC* participating in low-light- and high-light-induced chlorophyll accumulation, respectively [43]. In the rice mutant (*pgl10*), *OsPORB* is downregulated, and Chl a and Chl b contents are significantly reduced [44]. Our research has revealed that the expression pattern of chlorophyll catabolic genes exhibits a pronounced activity during the S3 phase (Figure 5), paralleling the trend observed in bananas [45]. Among genes involved chlorophyll degradation, all three *PaO* genes showed the highest expression level (Figure 5), indicating the key role of *PaO* in regulating chlorophyll degradation. PaO is a critical enzyme catalyzing the open-ring cleavage of the chlorophyll porphyrin to form a linear tetrapyrrole derivative. Studies on the induction of Arabidopsis leaf senescence in vitro under dark conditions showed that the expression, protein level and enzyme activity of *PaO* are consistently upregulated. They have noted that *PaO* predominantly regulates the degradation of chlorophyll at the transcriptional level, highlighting its significant role in chlorophyll catabolism during leaf senescence [46]. Increases in *PaO* activity or the expression of related genes are detected during fruit ripening in broccoli [47] and litchi [48], indicating a close relationship between enhanced chlorophyll catabolism and *PaO*. Similarly, in ‘Zaosu’ pear fruit, *PaO* has been identified as the key gene responsible for chlorophyll loss and chloroplast degradation in the fruit peel [49]. Therefore, we propose that increasing the activity of *PaO* genes during ‘Danruo No. 1’ pomegranate ripening could be a potential approach to maintaining good fruit appeal.

In this study, we analyzed the transcription factors at different developmental stages of ‘Danruo No.1’ pomegranate, and obtained 21 positive regulatory transcription factor families that may regulate anthocyanin accumulation, including MYB, WRKY, bHLH, GRAS, etc., and AP2/ERF is the only defined negative regulator (Figure 6D). MYB has been reported in several plant species, including Arabidopsis [50], tomato [51], apple [52] and tropical fruit [53]. The MBW complex composed of R2R3-MYB TFs, bHLH TFs and WD40 plays a key role in regulating the expression of structural genes in anthocyanin and flavonoid pathways, although specific members of each transcription factor family may differ in different species [54]. It has previously been reported that R2R3-MYB can activate the promoter of proanthocyanidin synthesis genes and regulate anthocyanin accumulation in peach flowers [55]. The activity of pomegranate regulatory gene *PgWD40* is confirmed in Arabidopsis *ttg1-9* mutant and proves to be dependent on MYB function [56]. In addition, some transcription factors such as WRKY [57], MYB (sweet cherry [58], grape [59], and kiwifruit [60]) and NAC [61] are also involved in the regulation of flavonoid metabolism. The transcriptional regulation mechanism of carotenoid biosynthesis has been well studied in different species. NAC22 [62] and AP2/ERF [63] can regulate the accumulation of carotenoids, thereby inducing fruit coloring. In our study, by constructing a correlation network, we obtained 12 classes of transcription factors (MYB, C2C2, AP2/ERF, bZIP, LOB, etc.) that positively regulate carotenoid components and 10 classes of negatively regulated transcription factors, mainly HB, MYB, GARP, HSF, etc. (Figure 6D). In addition, 29 TFs positively correlated with chlorophyll and 13 TFs negatively correlated with chlorophyll were obtained, mainly including MYB, C2H2, AP2/ERF, LOB, TCP, Trihelix and so on (Figure 6D). It has been reported that NAC016 directly binds to the *SGR1* promoter and regulates chlorophyll degradation [64]. Recently, the role of MYB in activating key chlorophyll biosynthesis genes to regulate chlorophyll accumulation and chloroplast development has also been confirmed in kiwifruit and tomato [65,66]. In addition, MYB [67], PIF [68] and NYC [69] transcription factors have also been shown to be involved in the regulation of chlorophyll metabolism.

## 4. Materials and Methods

### 4.1. Plant Materials

‘Danruo No.1’ pomegranate fruits were collected from a commercial pomegranate orchard located in Dongfang City, Hainan Province, China. Nine mature trees similar in size and number of fruit were chosen, with three trees per biological replicate. At 45 (Stage 1, S1), 120 (Stage 2, S2) and 160 (Stage 3, S3) days after full bloom (DAFB), three fruits per tree, reaching a total of nine fruits per biological replicate, were harvested. After measuring the fruit peel color index using a portable colorimeter (LS170, Shenzhen Linshang Technology Co., Ltd., Shenzhen, China), fruit peel was collected, frozen in liquid nitrogen, and then stored at −80 °C for later use.

### 4.2. Measurement of Anthocyanin, Chlorophyll and Carotenoid Contents

Anthocyanin content was determined as described previously, with slight modifications [70]. Here, 0.2 g pomegranate peel samples were ground in liquid nitrogen, added to 1.2 mL of 1% HCl–methanol extraction solution (methanol:HCL = 99:1, *v*/*v*), and vortexed and extracted overnight at 4 °C in the dark. After centrifuging at 12,000 rpm for 10 min at 4 °C, the supernatant was used for anthocyanin detection. The absorbance was measured at 530 nm and 600 nm using a spectrophotometer (UV759, APL Instrument Co., Ltd., Shanghai, China). Anthocyanin content was expressed as the difference between absorbance values at 530 nm and 600 nm.

Chlorophyll and carotenoid contents were determined as described previously, with slight modifications [71]. Here, 0.2 g pomegranate peel samples were ground, added to 3 mL 95% ethanol, vortex-mixed and extracted for 3–5 min. After centrifuging at 12,000 rpm for 10 min, the supernatant was diluted 2 times and the absorbance values of chlorophyll a, chlorophyll b and carotenoids were observed at 665, 649 and 470 nm using a spectrophotometer (UV759, APL Instrument Co., Ltd., Shanghai, China), respectively.

### 4.3. RNA Extraction, Library Construction and RNA-Seq

Total RNA was extracted using the RNAprep Pure Plant Kit (Polysaccharides & Polyphenolics-rich) (Tiangen, Beijing, China) according to the manufacturer’s protocol. RNA quality and concentration were analyzed by 1.0% agarose gel and a NanoDrop Lite Spectrophotometer (Thermo Scientific, Waltham, MA, USA), respectively.

RNA-Seq libraries were constructed by Wuhan Metware Biotechnology (Wuhan, China). The first strand of complementary DNA (cDNA) was synthesized from the fragmented mRNA template and random oligonucleotide primers. Then, deoxynucleotide triphosphates (dNTPs) were used as raw materials to synthesize the second strand of cDNA, and the double-stranded cDNA fragment was purified and recovered. The sequencing adaptor was ligated by end repair and A tail. The PCR products were purified by AMPure XP beads and the library was constructed. The library was diluted to 1.5 ng/uL by Qubit 2.0 Fluorometer (Thermo Scientific, Waltham, MA, USA), and the quality of the constructed library was checked by an Agilent 2100 bioanalyzer (Agilent, Santa Clara, CA, USA). When the insert size reached the expected value, the effective concentration of the library was accurately quantified by qRT-PCR to ensure the quality of the library. Each library was sequenced by the Illumina HiSeq2000 platform after the library inspection was qualified (Illumina Inc., San Diego, CA, USA). Low-quality data of the raw reads were removed by Fastp software 0.23.2 version (https://github.com/OpenGene/fastp, accessed on 16 May 2024), and TopHat [72] (http://tophat.cbcb.umd.edu, accessed on 16 May 2024) was then used to map clean reads to the pomegranate genome (NCBI accession number: PRJNA324150) [73]. Cufflinks (https://cole-trapnell-lab.github.io/cufflinks/, accessed on 17 May 2024) was used to assemble the transcripts [72], and gene expression was calculated and exhibited as fragments per kilobase of transcript per million of fragments mapped (FPKM). Differential expression analysis between two groups was performed using the DESeq2 v1.22.1 [74], and genes with |log2 fold change| > 1 and a significant *p*-value < 0.05 were considered as differentially expressed genes (DEGs). The raw data of the RNA-seq were submitted to NCBI with the following ID number: PRJNA1110799.

The interaction between co-expression modules was assessed using the Weighted Gene Co-Expression Network Analysis (WGCNA) algorithm. The concentrations of anthocyanins, chlorophyll and carotenoid in pomegranate peel during different developmental stages and all the expressed genes detected by RNA-seq (25,609 genes) were used for WGCNA. Highly correlated gene modules were identified using WGCNA in Metware Cloud (https://cloud.metware.cn/, accessed on 20 May 2024), and the results were visualized. The modules were built by the automatic network construction function ‘blockwise’. The mergeCutHeight and minModuleSize were set to 0.25 and 50, respectively. The characteristic gene values were calculated for each module and used for testing the association with each sample or trait. Using the module characteristic genes to estimate the module–trait relationship, the samples were classified according to the corresponding traits, and the module with a |correlation coefficient| ≥ 0.8 and *p*-value ≤ 0.01 was selected for further analysis. K-means clustering was performed for all differential genes in Metware Cloud (https://cloud.metware.cn/, accessed on 21 May 2024). In total, 8193 genes with suitable FPKM values were used to construct K-means clustering. The K-means clustering was obtained using the default parameters. Kyoto Encyclopedia of Genes and Genomes (KEGG, https://www.genome.jp/kegg/, accessed on 22 May 2024) [75] and Gene Ontology (GO, https://www.geneontology.org/, accessed on 22 May 2024) [76] databases were selected for gene function annotation. All heatmaps were generated using TBtools, and the z-score normalization function was used to standardize the data between rows [77].

### 4.4. cDNA Synthesis and q-PCR

First-strand cDNAs were synthesized from 1 μg total RNA, and reverse transcription was performed using the HiScript III 1st Strand cDNA Synthesis Kit (+gDNA wiper) kit (vazyme, R312-01, Nanjing, China). For q-PCR assays, the cDNA templates were diluted 20 times to provide appropriate template concentration. The primers were designed using Primer3web version 4.1.0 (https://primer3.ut.ee/, accessed on 28 May 2024) and had optimum melting temperatures (Tm) and GC contents. Subsequently, the newly designed primers were checked for specificity using Tbtools, ensuring their suitability for further experimental applications. The designed gene-specific primers are listed in Supplementary Table S4. q-PCR was performed in a real-time fluorescent quantitative PCR instrument (qTOWER3 G, Jena, Germany). The quantitative program was set up as described previously [78]. The data were normalized using the cycle threshold (Ct) value corresponding to the pomegranate *actin* gene to minimize variation in the level of cDNA templates. Relative expression was calculated for each target gene as described by Livak and Schmittgen [79]. Analysis was performed in three biological replicates.

### 4.5. Data Analysis

SPSS 27.0 (SPSS Inc., Chicago, IL, USA) was used to analyze all data via a one-way Analysis of Variance (ANOVA), and the mean values were separated by Tukey test for multiple comparisons. The probability value of <0.05 was considered statistically significant. Cytoscape software (version 3.10.2) was used to visualize the network relationship.

## 5. Conclusions

During the development of ‘Danruo No.1’ pomegranate fruit, the color of fruit peel turns from green to red, with constant increases in the a* value (representing redness) and anthocyanin concentration. In addition, the chlorophyll and carotenoid contents showed a decreasing pattern, and a decrease–increase pattern, respectively. Through RNA-seq, 8193 DEGs were identified, and KEGG enrichment analysis showed that flavonoid biosynthesis, carotenoid biosynthesis and phenylpropanoid biosynthesis were the most enriched pathways. Totals of 30, 18, and 17 structural genes related to anthocyanin biosynthesis, carotenoid biosynthesis and chlorophyll metabolism were screened from DEGs, respectively. Combined with WGCNA and K-means analyses, 45/1, 30/16, and 231/22 regulatory genes (encoding TFs) were identified as key candidate genes, which positively/negatively regulate the accumulation of anthocyanins, carotenoid, and chlorophyll, respectively, including MYB, WRKY, bHLH, AP2/ERF, bZIP, etc. The results of this study can provide a reference for the molecular mechanism of fruit peel color formation in evergreen pomegranate.

## Figures and Tables

**Table 1:** Summary statistics of RNA-seq data quality for 9 samples.

Sample	Raw Reads	Clean Reads	Clean Base (G)	Error Rate (%)	Q20 (%)	Q30 (%)	GC Content (%)
S1-1	52,424,972	51,682,184	7.75	0.03	97.95	94.01	49.81
S1-2	51,211,690	50,438,940	7.57	0.03	97.89	93.9	49.87
S1-3	53,622,346	52,811,144	7.92	0.03	97.86	93.82	49.9
S2-1	57,381,414	55,067,352	8.26	0.03	97.99	94.41	50.65
S2-2	49,502,618	48,759,030	7.31	0.03	97.86	93.81	49.95
S2-3	53,041,172	50,933,314	7.64	0.03	97.94	94.33	50.94
S3-1	45,436,078	44,890,314	6.73	0.03	97.96	94.02	50
S3-2	54,455,452	52,331,886	7.85	0.02	98.06	94.52	50.57
S3-3	42,335,952	41,696,868	6.25	0.03	98.01	94.12	49.91

## Data Availability

The data are contained within the manuscript and Supplementary Materials. The raw data of the RNA-seq were submitted to NCBI with the following ID number: PRJNA1110799.

## Supplementary Materials

The following supporting information can be downloaded at: https://www.mdpi.com/article/10.3390/plants13202903/s1, Figure S1: Principal component analysis of 9 samples; Figure S2: Go functional annotation of DEGs; Table S1: Results of one-way ANOVA analysis for the data; Table S2: Expression patterns of anthocyanin biosynthesis pathway, carotenoid biosynthesis pathway, and chlorophyll biosynthesis- and degradation-related differential genes; Table S3: Expression patterns of anthocyanin biosynthesis pathway and carotenoid biosynthesis pathway, and chlorophyll biosynthesis- and degradation-related differential transcription factor genes; Table S4: qRT-PCR primers of genes in pomegranate.
