# Supplementary figures and images for: Transcriptomic Analysis Reveals the Mechanism of Color Formation in the Peel of an Evergreen Pomegranate Cultivar ‘Danruo No.1’ During Fruit Development
Source: Plants (Basel). 2024 Oct 17;13(20):2903. doi: 10.3390/plants13202903 (PMC11511302; doi:10.3390/plants13202903)

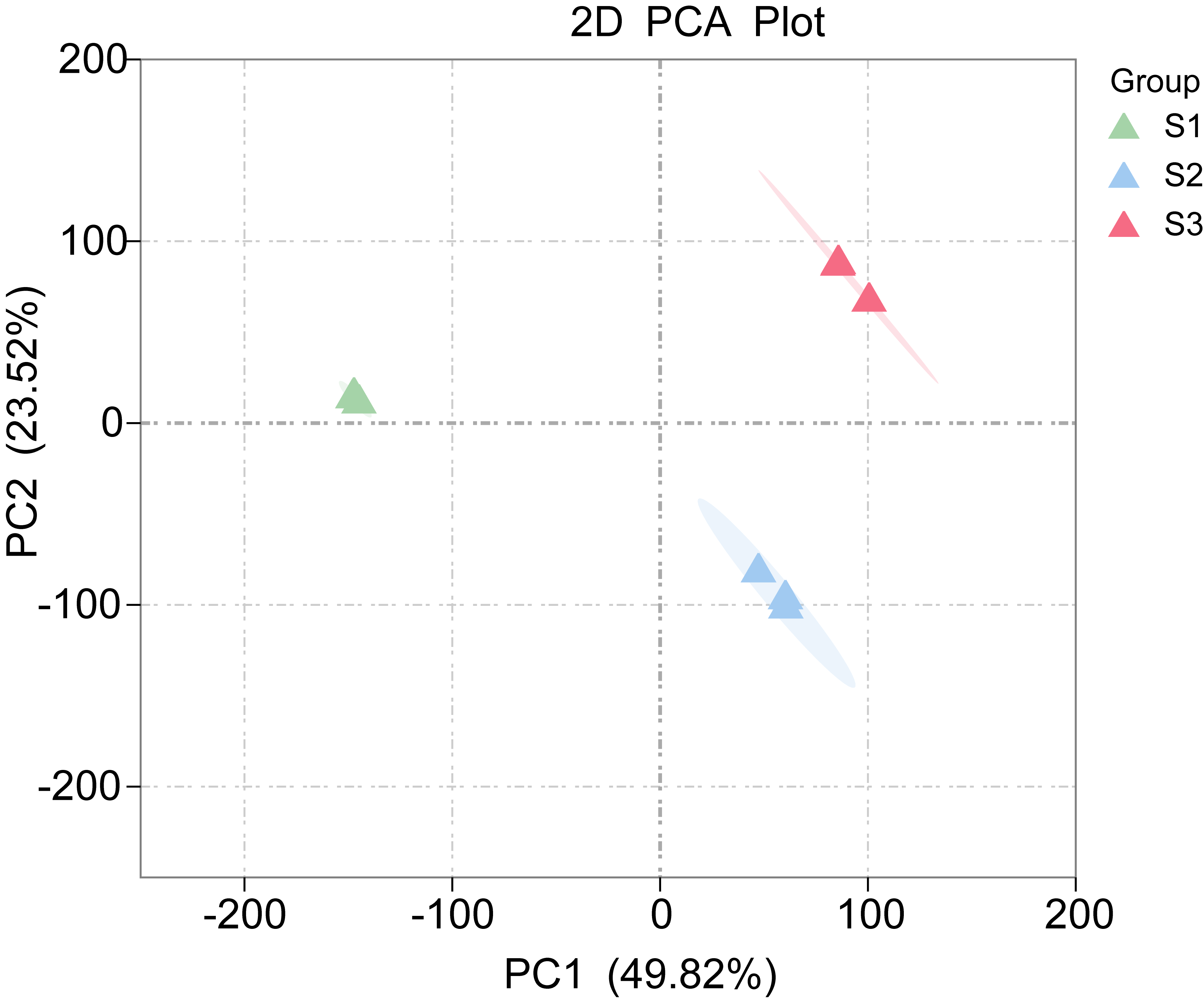

Supplement: Supplementary file 1 [file plants-13-02903-s001.zip › Figure S1.tif]
